# Good learners or trouble makers? Study on the relationship between academic performance and antisocial behavior of junior high school students

**DOI:** 10.1371/journal.pone.0295705

**Published:** 2024-01-02

**Authors:** Xiaobo Zhu, Wenyi Jiang, Weijin Shi, Junfeng Shi

**Affiliations:** 1 Faculty School of Finance and Business, Shanghai Normal University, Shanghai, P.R. China; 2 Lecturer Department of Basic, Shanghai Vocational College of Agriculture and Forestry, Shanghai, P.R.China; 3 School of Sport, Shanghai Normal University, Shanghai, P.R. China; 4 Department of Teacher Training, Huangpu Institute of Education, Shanghai, P.R. China; Nanjing Audit University, CHINA

## Abstract

The school bullying incident has aroused widespread concern in current society. How to manage students’ anti-social behavior has become an increasingly serious problem for administrators. This study uses a sample of 8270 junior high school students to examine the mechanism of academic achievement on students’ antisocial behavior. The results showed that academic performance has a U-shaped impact on antisocial behavior. This study further found that the U-shaped effect of academic performance on antisocial behavior was mediated by the praise; In addition, this study also found that moral identity moderates the U-shaped relationship between academic performance, praise, and antisocial performance. The findings provide the implications for school administrators and teachers to pay attention to the "moral trap" of academic achievement and praise, and pay attention to excellent students’ moral education, to reduce the possibility of their anti-social behavior.

## Introduction

A famous movie, titled ‘Better Days’, has aroused heated debate since it was released in October 2019 in China. One of the focuses of the debate was why Wei Lai, who had high academic performance, often committed anti-social behaviors, such as sneering at students, insulting verbally, and physically beating others. Indeed, the phenomenon that students with good academic performance violate social norms is common around us. For example, in 2017, a high-achieving student located in Hunan province in China stabbed his teacher to death because of the conflict with the teacher. In 2016, a 16-year-old student with good academic performance in Beijing assaulted a female classmate. High-achieving students’ bullying behavior at school was a serious issue and we hope students keep physical and psychological health. Wei Lai in the movie and the students mentioned above had excellent academic performance, being concerned by teachers and envied by students, and such "good students" can also violate social norms and even break the laws. This phenomenon means that if the high-achieving students do something deviating from the accepted social norms could be more damaging because both teachers and parents tend to be more tolerant of these students. How to avoid such a phenomenon has attracted the attention of related parties (e.g., administrators, students, parents, etc.). Therefore, in the background of "achievement is everything" in China, it is more important to understand how to induce students’ negative behaviors brought by excellent achievements.

While existing literature has explored academic performance and antisocial behavior independently [1, 2], there exists a notable gap in the research when it comes to investigating when and how academic performance is curvilinear related to antisocial behavior. This study endeavors to bridge this gap by examining how and when academic performance is U-shaped related to antisocial behavior. According to the moral licensing theory [3], the present study will explore the potential factors that may improve students’ antisocial behavior and the boundary conditions that will buffer the negative relations between excellent academic performance and antisocial behaviors, to provide theoretical support for preventing excellent students to behave anti-socially. Specifically, the inclusion of academic performance as the independent variable is substantiated by extensive research linking educational achievements with various social outcomes. The U-shaped relationship signifies that both extremely high and low academic performance levels can lead to increased antisocial behaviors. High achievers might engage in antisocial activities due to their praise. And moral identity is crucial in understanding how individuals navigate conflicting situations, particularly concerning their behavior. A strong moral identity can act as a buffer against antisocial tendencies, reinforcing the importance of aligning actions with moral principles. In summary, by examining the U-shaped relationship between academic performance of junior high school students and their antisocial behaviors contributing valuable insights to the existing body of knowledge in the field.

The primary objective of this study is to discern whether there exists a significant curvilinear correlation between the academic achievements of students and their engagement in antisocial behaviors. The research aims to provide valuable insights into understanding the behavioral dynamics of junior high school students, shedding light on whether good learners tend to exhibit more antisocial tendencies. And this study at least has three contributions. First, this study explores the relationship between academic performance and antisocial behavior, which can help us realize that the student with high performance may be related to antisocial behavior. Second, the present study investigates the mediating effect of praise in the inverted U-shaped relationship between academic performance and antisocial behavior, which can help us to understand how student’s academic performance is related to antisocial behavior. Third, our study also explores the boundary condition in which the academic performance will drive antisocial behavior, which can help us recognize when students with high academic performance will behave antisocial behavior.

## Literature review and research hypotheses

### Academic performance and antisocial behavior

Antisocial behavior refers to actions by individuals that violate societal norms, potentially causing harm or damage to others [4]. Previous research has consistently demonstrated a negative correlation between junior high school students’ academic performance and antisocial behavior. Specifically, poor academic performance is often associated with antisocial tendencies, and in some cases, it can effectively predict the emergence of antisocial behavior [1, 2, 5–7].

A meta-analysis investigating the relationship between academic performance and antisocial behavior highlighted that subpar academic achievements are strongly linked to the frequency, persistence, and severity of antisocial conduct. Conversely, excellent academic performance has been found to act as a deterrent against antisocial behavior [5]. Poor academic performance can lead to a decline in students’ self-esteem, subsequently diminishing their commitment to school, which, in turn, might contribute to criminal activities and antisocial behavior [7–9]. Therefore, when students with good academic performance have high self-esteem and a sense of superiority, they will show the self-perception of "excellent students" and restrain or avoid antisocial behavior. Although existing research is conducted from different theoretical perspectives or analytical levels, these studies share the premise that poor performance of students leads to antisocial behavior. Poor academic performance can lead to a decline in students’ self-esteem, subsequently diminishing their commitment to school, which, in turn, might contribute to criminal activities and antisocial behavior. Consequently, students with good academic performance, high self-esteem, and a sense of superiority tend to perceive themselves as “excellent students,” thereby restraining or avoiding antisocial behavior.

However, several crucial issues remain unaddressed. Specifically, it is essential to examine the potential hidden costs associated with exceptional academic performance in schools. Some high-achieving students engage in antisocial behavior, causing harm to their peers and negatively impacting the school environment. This phenomenon challenges the prevailing assumption that high performers always contribute positively to organizations. For example, Gino and Ariely [10] contested this assumption, demonstrating that high performers might be inclined to unethical behavior due to their tendency to justify such actions. They found that good performers were likely to engage in unethical behaviors since they were more likely to justify their unethical behaviors.

Moreover, research has shown that individuals with outstanding performance are often accompanied by feelings of pride and entitlement, which are significant predictors of antisocial behaviors [11–13]. For example, Vincent and Kouchaki [11] and Yam and Klotz [14] found that individuals who excel or possess remarkable abilities within a group tend to exhibit psychological entitlement and this sense of entitlement may drive them to engage in unethical behavior, further emphasizing the potential link between exceptional academic performance and increased antisocial behavior [12, 14]. In light of these findings, this study asserts that exceptional academic performance might lead to a rise in antisocial behavior.

The moral licensing theory provides a theoretical perspective to explain this phenomenon. The theory proposes that the behaviors that meet social expectations will induce future behaviors that deviate from social norms [15, 16]. Behaviors that meet social expectations will improve the individual’s moral self-perception, and they conduct “bad things” after exceeding the balance point of self-moral perception [17–19]. This provides a theoretical perspective for us to explain why students with high academic performance engage in antisocial behavior. Academic performance meets the expectations of society, schools, and parents, which will bring positive feedback to the students [6]. These positive feedbacks, such as praise, will improve students’ self-esteem [20], and self-esteem can effectively reduce antisocial behavior [21]. According to the moral licensing theory, this kind of self-moral cognition brought by academic achievements will allow students to engage in antisocial behavior and will not be punished.

Bandura [22] suggests that prosocial behavior is regulated by two major types of sanctions: social sanction and internalized sanction, both of which operate in an anticipatory way. As most antisocial behaviors go socially undetected, social sanctions have limited deterrent power. Thus, internalized sanctions play a central role in regulating moral behaviors [22, 23]. Individuals do not conduct immoral or antisocial behaviors unless they can find reasons to justify these behaviors, the process of justification is called moral disengagement. From the perspective of moral licensing theory, when individuals conduct the behavior or obtain outcomes that meet social expectations, they will decrease their moral standards and are more likely to conduct behaviors that violate social norms (e.g., [24]). Also, Mazar, and Amir [25] reveal that when an individual achieves excellent performance expected by society, he will forget moral norms, and improve the possibility of engaging in antisocial behaviors. Studies have found that when an individual engages in prosocial behaviors expected by the society, he/she is more likely to lie and cheat [25]. In line with this logic, the student with high academic performance always obtains high academic performance that meets social and parents’ expectations, these excellent students may decrease their requirements of moral standards, and conduct behaviors that deviate from social norms. Further, excellent academic performance will lead to students’ pride and psychological entitlement [12, 14]. Pride and psychological entitlement are powerful predictors of individual deviant behavior. Therefore, student surrounded by praise and approved, they will conduct more bullying at school since they have psychological entitlement that makes them believe that they will not be punished even if they conduct antisocial behaviors. In line with what we have discussed above, we propose hypothesis 1.

**Hypothesis 1:** There is a U-shaped relationship between academic achievement and antisocial behavior.

### The mediating role of praise

Scholars suggested that the effect of academic performance on antisocial behavior may be through a mediating mechanism [2]. However, few scholars have empirically tested mediators to explain the relationship between student’s academic performance and their antisocial behavior [6]. When students have excellent academic performance, they always attract the attention of teachers and get more praise from their teachers [26, 27]. Teachers’ praise will strengthen students’ perception that their academics meet the teacher’s expectations and make them loosen their moral standards, which in turn are likely to engage in antisocial behaviors. Furtherly, some scholars have pointed out that when individuals are excessively praised, that may produce negative results [28], such as the reduction of intrinsic motivation [29]. As mentioned above, personal entitlement is often accompanied by the emergence of antisocial behavior [12, 14]. Drawing on what we have discussed above, we propose Hypothesis 2.

**Hypothesis 2a:** There is a U-shaped relationship between praise and antisocial behavior.
**Hypothesis 2b:** Praise mediates the U-shaped relationship between academic performance and antisocial behavior.

### The moderating role of moral identity

Many students who have academic performance do not necessarily engage in antisocial behaviors, although students who have achieved academic success will be likely to lead to antisocial behaviors [30]. Therefore, several scholars have proposed that the relationship between academic performance and antisocial behavior is moderated by boundary conditions [31, 32]. Whether the moral licensing brought by academic achievements and teachers’ praise promotes them to engage in antisocial behavior depends on an individual’s moral sensitivity. A few studies have pointed out that moral identity is an individual difference variable that relates to moral sensitivity [33, 34]. Moral identity reflects the individual’s recognition of moral issues such as fairness, caring for others, and loyalty [35]. Moral identity schema affects individuals’ interpretation of social situations [36]. Individuals with high moral identity are more inclined to activate the schema of moral self-regulation, attach importance to self-moral commitment, inhibit cognition and behavior that violate social norms, and maintain the consistency between behavior and social moral norms [37]. In line with the definition of moral identity, research shows that students with high moral identity make decisions in conducting behaviors from the perspective of social moral norms [38, 39]; therefore, such traits will inhibit the negative effects of moral license. In other words, even if academic achievements and praise will bring students a sense of moral license, the moral identity schema will determine their future behavior decisions. Specifically, they will conduct their behaviors according to moral standards. Therefore, with the increasing academic achievement, students with high moral identity will not necessarily behave anti-socially. However, for the individual with low moral identity, moral standards does not play important role in the process of decision-making, self-moral schema will not be activated by the situation, and they are less likely to make behavioral decisions from the perspective of social moral norms [37]. In line with this rationale, students seldom make decisions on conducting behaviors from the perspective of internal moral norms. Since the entitlement or moral license brought by academic achievements and praise, students will feel more comfortable engaging in antisocial behaviors. Thus, when students’ moral identity is low, the relationship between academic achievement or praise and antisocial behavior is still U-shaped. Drawing on what we have discussed above, we propose Hypothesis 3.

**Hypothesis 3a:** Moral identity moderates the curvilinear effect of academic achievement on antisocial behavior. Specifically, when the student holds a low level of moral identity, the U-shaped relationship between academic performance and antisocial behavior is steeper than the one who holds a high level of moral identity.
**Hypothesis 3b:** Moral identity moderates the curvilinear effect of praise on antisocial behavior. Specifically, when the student holds a low level of moral identity, the U-shaped relationship between academic performance and antisocial behavior is steeper than the one who holds a high level of moral identity.

**Fig 1:**
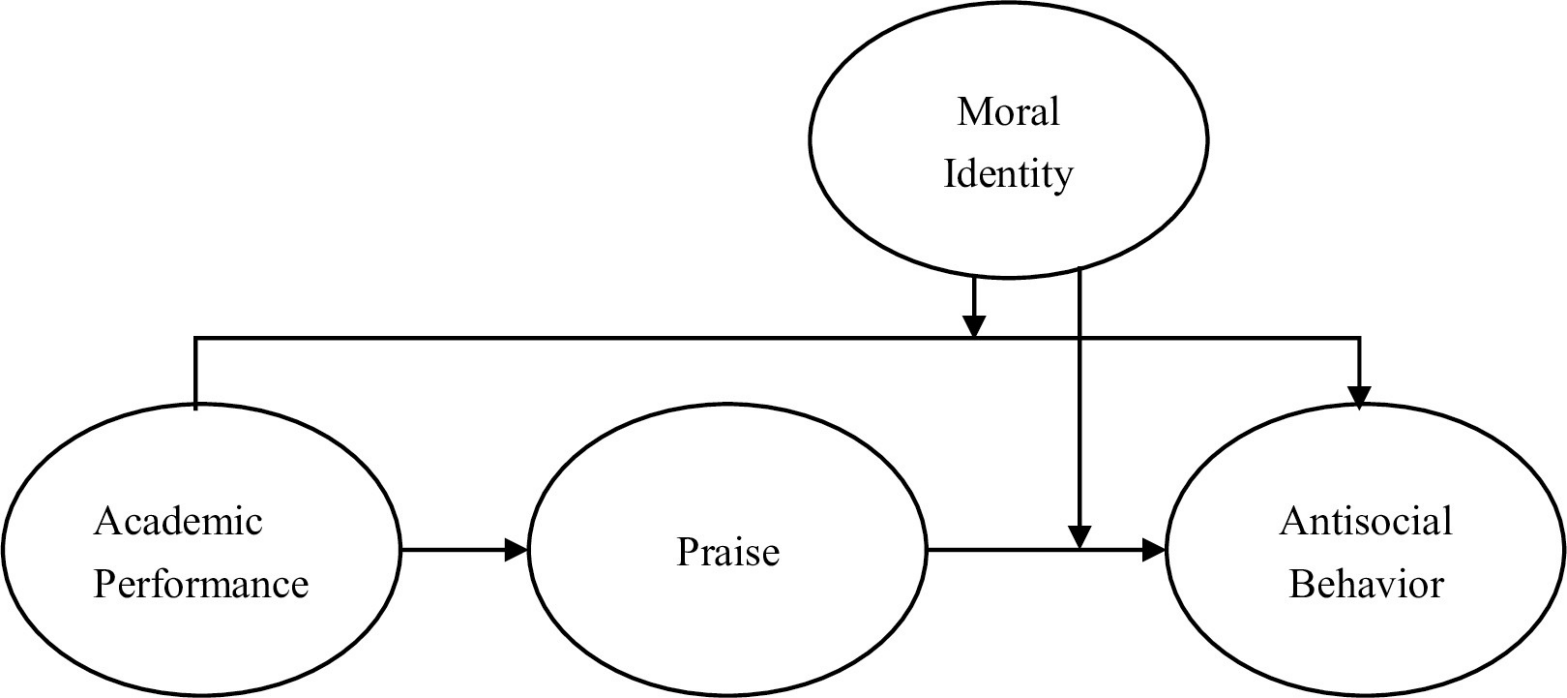
Research model.

Following what has been proposed above, this study presents the research model in Fig 1.

## Methods

### Samples and data

The data was collected by the National Survey Research Center (NSRC) at Renmin University of China, which conducted a program titled China Education Panel Survey (CEPS) to explore the psychological status and learning performance of junior students. Given the data was opened for research scholars, we confirm that all aspects of this research were conducted in compliance with ethical standards and guidelines for secondary data analysis, ensuring the confidentiality and privacy of the participants involved in the original surveys.

The survey contains two waves of sample collecting. The first wave was conducted in the 2013–2014 academic year of Grade 7 of junior high schools. A total of 10,279 samples were randomly collected from 438 classes located at 112 schools. In the second survey, conducted in the 2014–2015 academic year, 9449 students (rate of response is 91.9%) were successfully traced and completed the questionnaire. After matching the two datasets and deleting the missing values of related variables, 8270 valid samples were retained in this study. In the current study, except the gender, all the other variables came from the second-wave survey. In addition, it is generally believed that Chinese, mathematics, and English are the three main subjects in the study of junior high school in China. Therefore, we consider the performance of the three subjects to be an index to measure academic performance.

### Measurement

#### Academic performance

Although the CEPS survey did not directly measure the students’ self-reported academic performance, the survey measured the oppressive feelings experienced by students in the study of a certain subject. Generally speaking, the less oppressive feelings experienced by students, the better their academic performance is often [40]. Therefore, this study uses "Do you feel difficult to learn mathematics now?" "Do you find it difficult to learn Chinese now?" "Do you find it difficult to learn English now?" Three questions are used to characterize junior high school student’s academic achievements, and the four-point scale method is used to score (1 = "particularly difficult", 2 = "a little difficult", 3 = "not very difficult", 4 = "not difficult at all"). Although there is a little mismatch between concept and measurement, there are many studies that suggest perceived difficulty in the processes of learning is strong and negatively related to academic performance [40], it means that perceived difficulty is smaller, the academic performance is better. Therefore, we believe that it can, at least to some extent, to measure academic performance.

To decrease the potential threat of this issue on the measurement of academic performance, this study further uses the academic performance reported by parents (your child’s current performance in the class is: 1 = bad, 2 = middle and small, 3 = medium, 4 = middle and upper, 5 = very good) to carry out robustness test to examine the stability of the findings in this study. This measurement directly measures the academic performance of students, which can reflect the degree of academic performance.

The Cronbach coefficient of the measuring tool is 0.60, which may be due to the uneven development of students in Mathematics, Chinese, and English, resulting in a low coefficient. However, the value also meets the minimum critical value proposed by scholars [41].

#### Praise

We use the students’ perceived praise from mathematics ("mathematics teacher often praises me"), Chinese ("Chinese teacher often praises me"), and English ("English teacher often praises me") teachers in CEPS, and the 4-point scale method is used to rate the praise (1 = "totally disagree", 2 = "relatively disagree", 3 = "relatively agree", 4 = "totally agree"). The Cronbach coefficient of the three questions on praise measurement is .88.

#### Moral identity

In line with the definition of moral identity-reflecting the individual’s recognition of moral qualities such as fairness, caring for others, and loyalty [42], Aquino and Reed [38] also argue that fairness, friendly, and helping are the key traits of the individual with high level of moral identity. Therefore, this study uses CEPS’s three items "obeying order, consciously queuing up" (fairness), "helping the elderly to do things" (helping), and "being sincere and friendly to others" (friendly) to measure moral identity, and the three items are rate by the 5-point scale (1 = "never", 2 = "occasionally", 3 = "sometimes", 4 = "often", 5 = "always"). The Cronbach coefficient of these three questions on moral identity measurement is .67.

#### Antisocial behavior

Based on the measurement of adolescent antisocial behavior developed by Achenbach and Edelbrock [4], this study uses CFPS’s 7 items (e.g., "swearing ", "quarreling", "fighting", "bullying weak classmates", "skipping classes, truancy ", "smoking, drinking", "going to net cafes, game halls") to measure students’ antisocial behavior, and the seven items are rated by the 5-point scale (1 = "never", 2 = "occasionally", 3 = "sometimes", 4 = "often", 5 = "always"). To control the common method bias in this study and test the robustness of our findings, this study uses the antisocial data in the parents’ questionnaire and the students’ self-rating questionnaire in the CFPS survey for hypothesis testing and robustness testing. The Cronbach coefficients of antisocial behavior measurement tools of parents’ evaluation questionnaire and students’ evaluation questionnaire are .77 and .79, respectively.

#### Control variables

Several studies have pointed out that family relationships affect students’ antisocial behavior [6, 43]. Therefore, this study focuses on the relationship between parents (0 = "good", 1 = "bad"), the relationship with the father (1 = "not close", 2 = "average", 3 = "very close"), and the relationship with mother (1 = "not close", 2 = "average", 3 = "very close") A review study suggests that the socio-economic status of the family may affect students’ antisocial behavior [44]. This study controls this potential factor and uses family economic conditions to measure this variable (1 = "very difficult", 2 = "relatively difficult", 3 = "medium", 4 = "relatively rich", 5 = "very rich"). In addition, studies have shown that students’ background characteristics may be related to antisocial behavior [6, 9, 44]. This study controls students’ gender (0 = "male", 1 = "female"), household registration place (0 = "local county (district)", 1 = "other county (district)"), and whether they are only children in a family (0 = "yes", 1 = "no").

### Data analysis method

In this study, we use regression analysis to test the curvilinear effect of academic achievement and praise on antisocial behavior. The mediating effect of praise is tested by the approach proposed by Hayes and Preacher [45]. because the mediating effect of this study is nonlinear, the traditional mediating test method is not suitable for the content of this study. This study uses the approach proposed by Aiken and West [46] to test the moderating effect of moral identity.

## Results

### Descriptive statistics and correlation analysis

The description and correlation analysis tables of the data are shown in Tables 1 and 2 respectively. The results show that there is a negative correlation between academic performance, praise, and antisocial behavior (r = -0.21, p < 0.001; r = -0.10, p < 0.001), which indicates that the better the performance or the more praise received from the teacher, the students will conduct lower antisocial behavior. Therefore, whether academic performance and praise will change this relationship after reaching a certain level needs further analysis.

**Table 1:** Sample description (N = 8270).

Variable	Measurement	Frequency	Proportion
Gender	0 = male	4234	51.20%
	1 = female	4036	48.80%
Registration	0 = Local	6794	82.15%
	1 = Other	1476	17.85%
One child/family	0 = Yes	3749	45.33%
	1 = No	4521	54.67%
Economic situation	1 = Very difficult	221	2.67%
	2 = Difficult	991	11.98%
	3 = Medium	6085	73.58%
	4 = Rich	916	11.08%
	5 = Very rich	57	0.69%
Parental relationship	0 = Good	7450	90.08%
	1 = Not good	820	9.92%
Relationship with father	1 = Not close	343	4.15%
	2 = General	3369	40.74%
	3 = Very close	4558	55.11%
Relationship with mother	1 = Not close	160	1.93%
	2 = General	2068	25.01%
	3 = Very close	6042	73.06%

**Table 2:** Mean, standard deviation and correlation coefficient.

	1	2	3	4	5	6	7	8	9	10	11	12	13
1.Gender													
2.Registration	-0.03*												
3.One child/family	0.06***	0.12***											
4.Economic situation	0.00	0.04**	-0.19***										
5.Parental relationship	0.02*	-0.01	-0.02	-0.10***									
6.Relationship with father	-0.02	-0.04***	-0.06***	0.07***	-0.30***								
7.Relationship with mother	0.06***	-0.05***	-0.06***	0.08***	-0.24***	0.45***							
8.PAP	0.16***	-0.01	-0.03**	0.06***	-0.06***	0.11***	0.11***						
9.SAP	0.14***	0.01	-0.19***	0.25***	-0.09***	0.17***	0.17***	0.43***	(0.60)				
10.Praise	0.01	-0.02*	-0.08***	0.08***	-0.10***	0.20***	0.18***	0.19***	0.24***	(0.88)			
11.Moral identity	0.10***	-0.02	-0.09***	0.08***	-0.07***	0.17***	0.18***	0.16***	0.24***	0.20***	(0.67)		
12.PAB	-0.19***	0.03*	0.12***	-0.05***	0.09***	-0.13***	-0.15***	-0.23***	-0.21***	-0.10***	-0.19***	(0.77)	
13.SAB	-0.21***	0.04**	0.08***	-0.04***	0.11***	-0.15***	-0.17***	-0.21***	-0.22***	-0.13***	-0.26***	0.47***	(0.79)
Mean value	.49	.18	.55	2.95	1.10	2.51	2.71	3.10	2.56	2.39	3.81	1.25	1.41
SD	0.50	0.38	0.50	0.60	0.30	0.58	0.49	1.05	0.65	0.85	0.76	0.34	0.44

Note: * p <. 05, ** p <. 01, *** p <. 001; PAP = Parent rated academic performance, SAP = Self rated academic performance, PAB = Parent rated antisocial behavior, SAB = Self rated antisocial behavior; Values in diagonal and bold is Cronbach coefficient

### Hypothesis testing

We follow the suggestion proposed by Aiken and West [46] to avoid the collinearity among academic performance, the square term of academic performance and praise, and the interactive term between academic performance and moral identity, the present study centralizes academic performance, praise, and moral identity to generate square term and interactive term.

**Fig 2:**
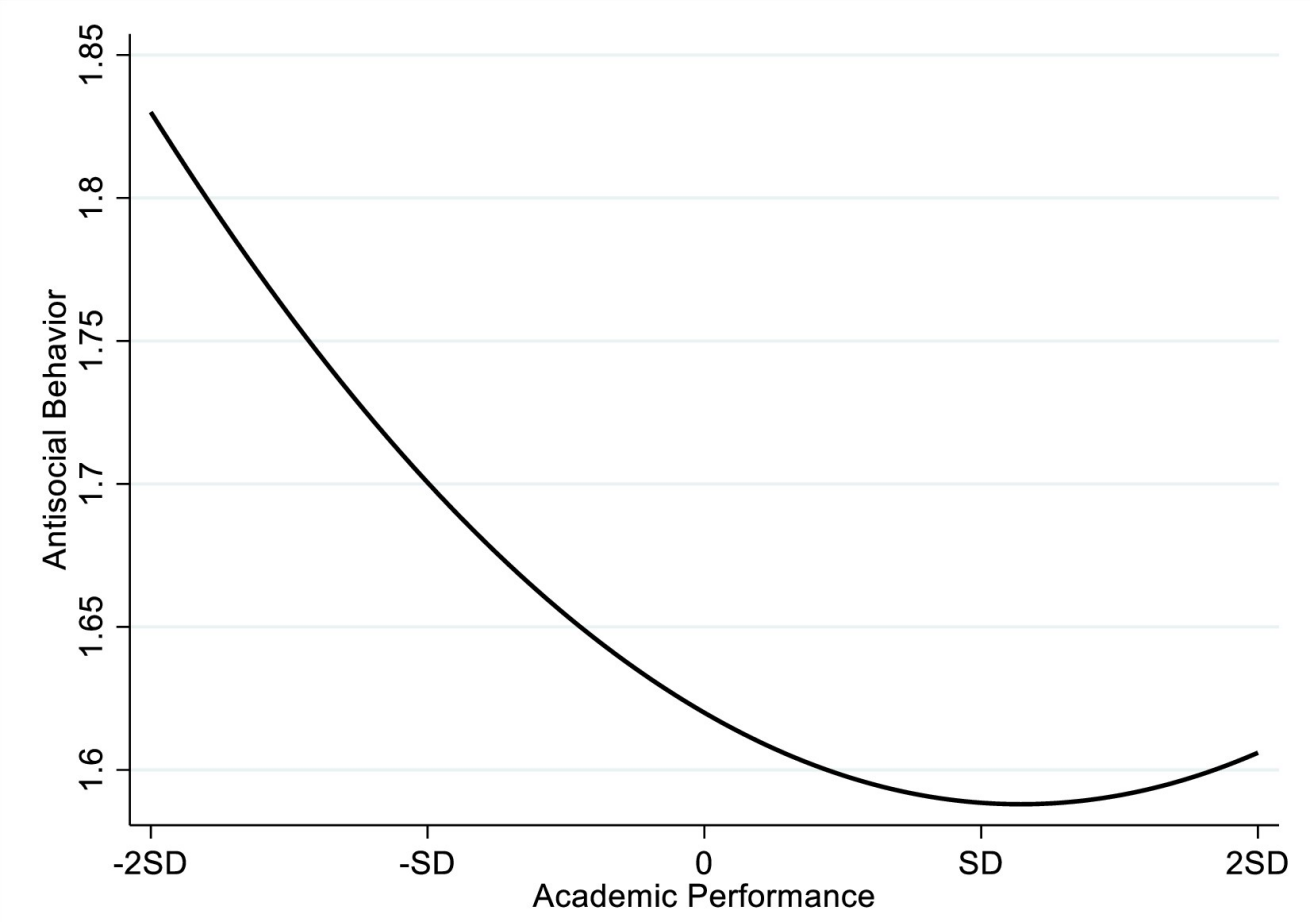
The U-shaped relationship between academic performance and antisocial behavior.

To test the hypothesis, the present study uses STATA 14.0 to analyze the data. The regression results are presented in Table 3. The results of Model 3 show that there is a positive correlation between the square term of academic performance and antisocial behavior (β = .08, p < .001), which indicates that there is a curvilinear relationship between academic performance and antisocial behavior. Fig 2 shows that there is a U-shaped relationship between academic performance and antisocial behavior. Specifically, the student’s antisocial behavior decreases with the increase in academic performance; however, when the academic performance exceeds a certain threshold, the student’s antisocial behavior will increase with the increase in academic performance. To further test whether the positive correlation between academic performance and antisocial behavior is significant or not when academic performance exceeds the threshold, the present study conducts the simple slope test of the curve according to the approach proposed by Aiken (46), and the results of simple slope test are shown in Table 4. The results suggest that when the academic performance was at-2SD,-SD, 0, there was a significant negative relationship between academic performance and antisocial behavior (β = -.210, p < .001; β = -.145, p < .001; β = -.080, p < .001); When academic achievement rises to 1 SD, academic performance is still negatively but not significant related to antisocial behavior (β = -.015, p > .050), which indicates that with the increasing of academic performance, its negative effect of academic performance on antisocial behavior is weakening; When the academic performance rises to 2 SD, academic performance has turned to be significant positively related to antisocial behavior (β = .050, P < .010). Thus, Hypothesis 1 is supported.

**Table 3:** Regression analysis.

Variable	Antisocial Behavior (rated by parents)								
	Model 1	Model 2	Model 3	Model 4	Model 5	Model 6	Model 7	Model 8	Model 9
Gender	-0.19 ***	-0.17***	-0.16***	-0.16***	-0.16***	-0.19***	-0.19***	-0.18***	-0.18***
Registration	0.00	0.01	0.01	0.01	0.01	0.00	0.00	0.00	0.00
One child/family	0.12***	0.10***	0.10***	0.09***	0.09***	0.11***	0.11***	0.11***	0.10***
Economic situation	-0.01	0.02	0.02	0.02	0.02	-0.01	-0.01	0.00	0.00
Parental relationship	0.05***	0.05***	0.04***	0.04***	0.04***	0.05***	0.05***	0.05***	0.05***
Relationship with father	-0.07***	-0.05***	-0.05***	-0.04***	-0.04***	-0.06***	-0.06***	-0.05***	-0.05***
Relationship with mother	-0.09***	-0.08***	-0.07***	-0.06***	-0.06***	-0.08***	-0.08***	-0.07***	-0.07***
AP		-0.14***	-0.14***	-0.12***	-0.12***				
AP^2^			0.08***	0.08***	0.08***				
DBP						-0.06***	-0.06***	-0.04***	-0.04***
DBP^2^							0.04***	0.04***	0.04***
Moral identity				-0.12***	-0.09***			-0.13***	-0.11***
AP*MI					0.00				
AP^2^*MI					-0.04**				
DBP*MI									-0.02
DBP^2^*MI									-0.03*
R^2^	0.08	0.09	0.10	0.11	0.12	0.08	0.80	0.09	0.10
F	98.09***	107.29***	102.19***	104.50***	87.70***	89.47***	80.97***	88.56***	74.56***

Note: * p < .05, ** p < .01, *** p < .001; AP = Academic performance, DBP = Degree of being performance

**Table 4:** Simple slope test of academic performance on antisocial behavior.

Academic performance	Slope	Slope variance	T value
-2SD	-0.210***	0.018	-11.471
-SD	-0.145***	0.011	-13.715
0	-0.080***	0.006	-13.457
SD	-0.015	0.010	-1.458
2SD	0.050**	0.018	2.781

Note: ** p < .01, *** p < .001

**Fig 3:**
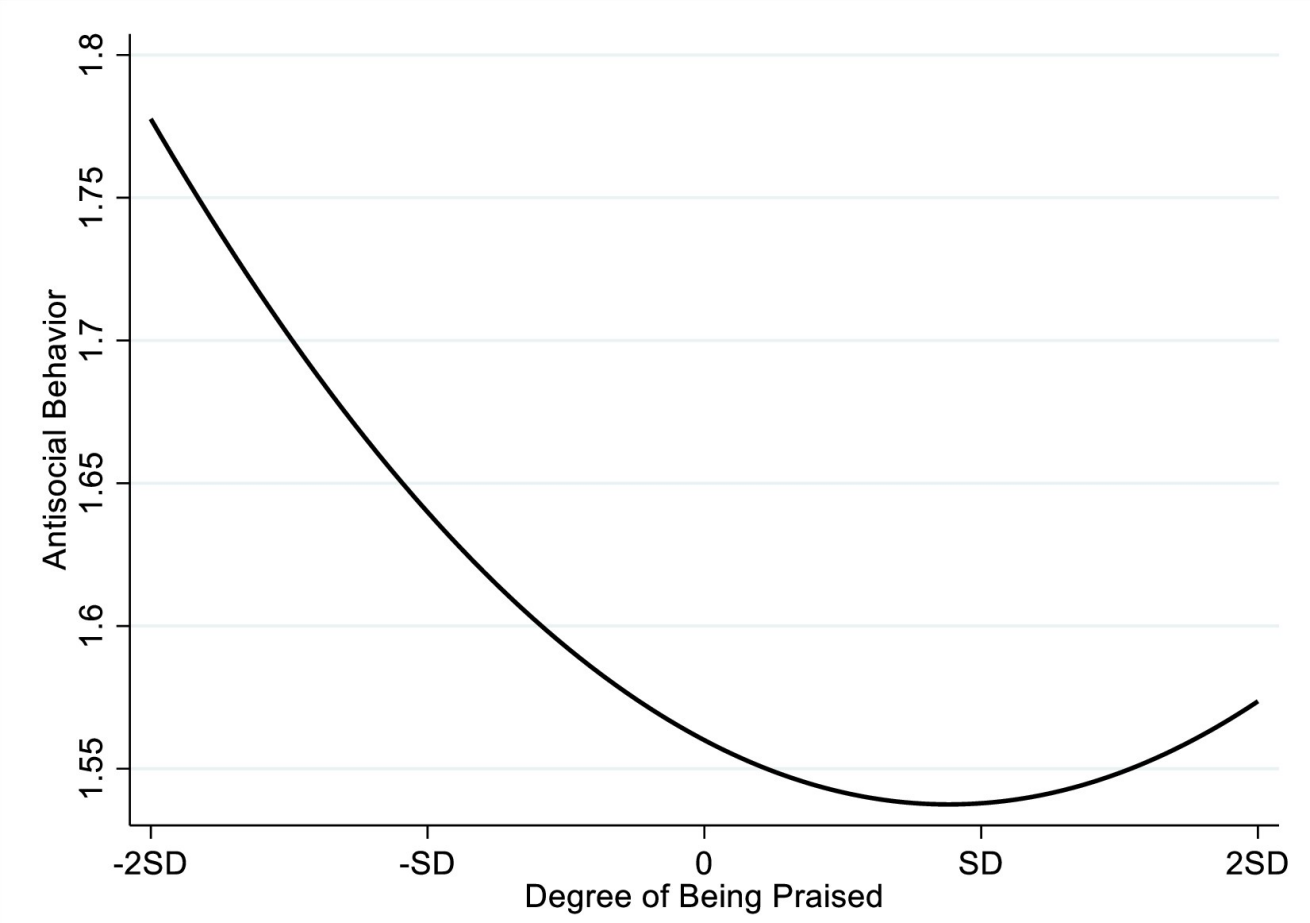
The inverted U-shaped relationship between the praise and antisocial behavior curve.

The regression results of Model 7 in Table 3 show that there is a positive correlation between the praise square and antisocial behavior (β = .040, p < .001), which indicates that there is a curvilinear relationship between praise and antisocial behavior. The results of the curvilinear relationship presented in Fig 3 show there is a U-shaped relationship between praise and antisocial behavior. Specifically, the student’s antisocial behavior decreases with the increase of the praise; however, when the praise exceeds a certain threshold, the student’s antisocial behavior will increase with the increase of praise. In the present study, we also used the method mentioned above to conduct the simple slope. The results of the simple slope test are shown in Table 5. The results suggest that when praise was at-2SD,-SD, 0, there was a significant negative relationship between the praise and antisocial behavior (β = -.161, p < .001; β = -.105, p < .001; = -0.050, p < 0.001); When the praise rises to 1 SD, the praise has a positive but insignificant relationship with antisocial behavior (β = .006, p > .050). Although the positive relationship is not significant, it indicates that it’s the negative effect on antisocial behavior is weakening until it disappears with the increase of the praise; When the praise rises to 2 SD, antisocial behavior has turned to be significantly positively related to the praise (β = .062, p < .010). Thus, Hypothesis 2a is supported.

**Table 5:** Slope test of the praise on antisocial behavior.

Praise	Slope	Slope variance	T value
-2SD	-0.161***	0.021	-7.812
-SD	-0.105***	0.012	-8.936
0	-0.050***	0.006	-8.711
SD	0.006	0.010	0.610
2SD	0.062**	0.019	3.277

Note: ** p < .01, *** p < .001

In order to further test the mediating effect of praise in the curvilinear relationship between academic performance and antisocial behavior, the present study adopts the methods proposed by Hayes and Preacher [45]. When there is a nonlinear relationship in the path that the independent variable (X) impacts on the dependent variable (Y) through the intermediate variable (M), the change rate of Y caused by the change of M due to the change of X is expressed by θ [47]. The θ can be calculated by the product of the first-order partial derivative of M for X and the first-order partial derivative of Y for M (Formula 1).


$$\theta =\frac{\partial M(X)}{\partial X}\frac{\partial Y(X,M)}{\partial M}$$ (1)

Hayes and Preacher [45] call this indirect change rate as an instantaneous mediating effect, and the value can be calculated by assigning a specific value to an independent variable (X), and then its significance can be examined by the Bootstrap approach. According to the steps proposed by Hayes and Preacher [45], the indirect effect and its confidence interval were calculated by the bootstrap approach in STATA14.0. Table 6 shows the instantaneous indirect effects of bootstrapping 1000, 2000, and 5000 repeated samples when the academic performance was assigned to -2SD, -SD, 0, 1SD, and 2SD.

**Table 6:** Instantaneous indirect effect of the praise.

Academic performance	Bootstrapping No.	Transient mediating effect	Standard deviation	Confidence interval	
				Lower	Upper
-2SD	1000	-0.046	0.007	-0.059	-0.032
-SD		-0.031	0.004	-0.039	-0.023
0		-0.016	0.002	-0.021	-0.012
SD		-0.002	0.003	-0.008	0.005
2SD		0.013	0.006	0.001	0.024
-2SD	2000	-0.046	0.007	-0.059	0.033
-SD		-0.031	0.004	-0.039	-0.023
0		-0.016	0.002	-0.021	-0.012
SD		-0.002	0.003	-0.009	0.005
2SD		0.013	0.006	0.001	0.024
-2SD	5000	-0.046	0.007	-0.059	0.033
-SD		-0.031	0.004	-0.039	-0.023
0		-0.016	0.002	-0.021	-0.012
SD		-0.002	0.003	-0.008	0.005
2SD		0.013	0.006	0.001	0.024

When the academic performance is located at -2SD, the instantaneous indirect effect is -.046, and the confidence interval does not include 0, which indicates that the instantaneous indirect effect is significant at this value. When academic performance is located at -1SD, the instantaneous indirect effect is -. 031, while the confidence interval does not include 0, indicating that the instantaneous indirect effect is significant at the value of -1SD. When academic performance is located at 0, the instantaneous indirect effect is -.014, but the confidence interval still does not include 0, indicating that the instantaneous indirect effect is significant at the value of 0. Similarly, when the level of academic performance rises to 1SD, the instantaneous indirect effect is -. 020, and the confidence interval includes 0, indicating that the instantaneous indirect effect is not significant at the value of 1SD. When the academic performance is located at 2SD, the instantaneous indirect effect is .013, and the confidence interval of this effect value does not include 0, which indicates that the instantaneous indirect effect is significant. These results show that the instantaneous indirect effect of praise between academic achievement and antisocial behavior changes from significant negative to significant positive with the increase in academic performance. Thus, Hypothesis 2b is supported.

**Fig 4:**
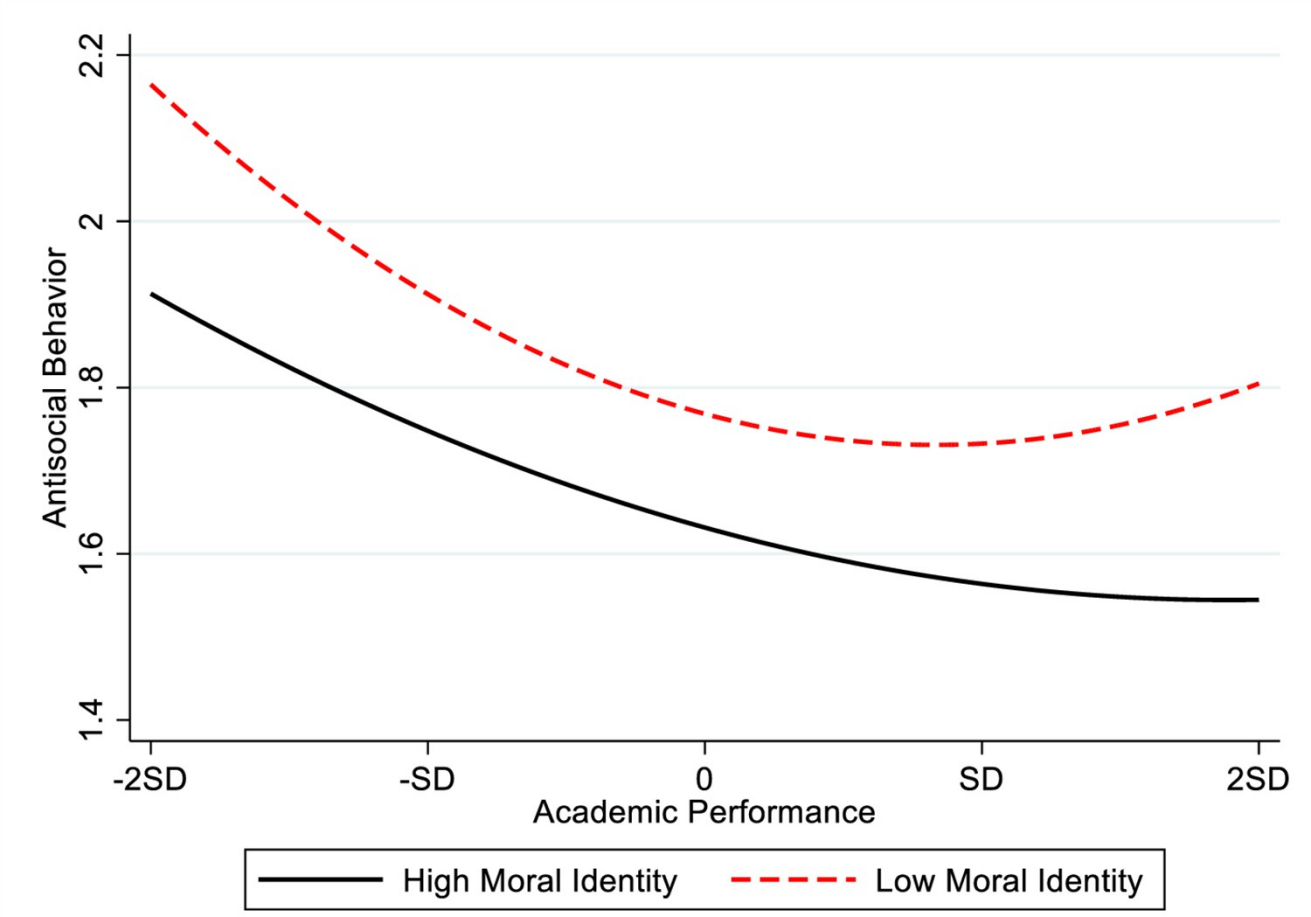
The moderating effect of moral identity on the curvilinear relationship between academic performance and antisocial behavior.

We used the approach proposed by Aiken and West [46] to test Hypothesis 3a and 3b, the results are shown in Table 3. Model 5 in Table 3 shows that the interactive term between academic performance square and moral identity is negatively related to antisocial behavior (β = -. 004, p < 0.01), which suggests that moral identity moderates the U-shaped relationship between academic performance and antisocial behavior. In order to clearly display the moderating effect of moral identity on the curvilinear relationship between academic performance and antisocial behavior, the present study draws the moderating effect of moral identity according to the regression coefficient (Fig 4). Fig 4 shows antisocial behavior presents a pattern of first falling and then rising with the increasing of academic performance when the student’s moral identity is low; however, there is a stable negative relationship between academic achievement and antisocial behavior when the student’s moral identity is high. In other words, the U-shaped relationship between academic performance and antisocial behavior is steeper when the student’s moral identity is lower. Thus, Hypothesis 3a is supported. Further, the present study conducts the simple slope test to test whether the impact of academic performance on antisocial behavior is significant or not in the different cases of moral identity, and the results are shown in Table 7. In the case of a high level of moral identity, academic performance is negatively related to antisocial behavior when academic performance located at-2SD,-1SD, 0, 1SD, and 2SD (β = -.103, p < .001; β = -.084, p < .001; β = -.066, p < .001; β = -.047, p < .001; β = -.028, p > .001). Although at the value of 2SD, the effect of academic performance on antisocial behavior is not significant (β = -.028, p > .050), it is still negative. It means that, with the increasing academic performance, its negative effect on antisocial behavior may be weakening when students’ moral identity is high. In the case of a low level of moral identity, academic performance is negatively related to antisocial behavior when the academic performance located at -2SD, -1SD, and 0 (β = -0.216, p < .001; β = -.139, p < .001; β = -.062, p < .001); however, when the academic performance rises to the value of 1SD, the academic performance has a positive but insignificant impact on antisocial behavior (β = .015, p > .050), and when the academic performance raise to the value of 2SD, there is a positive significant effect of academic performance on antisocial behavior (β = .092, P < 0.001). This shows that, when their academic performance exceeds a certain level, it will promote student conduct antisocial behavior in the condition of students with low moral identity, and this positive effect is stronger than the main effect (compared with the slope at 2SD in Table 4), Thus, Hypothesis 3a is supported.

**Table 7:** Simple slope test of academic performance* moral identity on antisocial behavior.

Adjustment variable	Academic achievement	Slope	Slope variance	T value
High moral identity	-2SD	-0.103***	0.023	-4.420
	-SD	-0.084***	0.014	-6.009
	0	-0.066***	0.008	-8.298
	SD	-0.047***	0.012	-3.981
	2SD	-0.028	0.021	-1.374
Low moral identity	-2SD	-0.216***	0.021	-10.422
	-SD	-0.139***	0.012	-11.706
	0	-0.062***	0.008	-7.934
	SD	0.015	0.014	1.049
	2SD	0.092***	0.023	3.973

Note: *** p < .001

**Fig 5:**
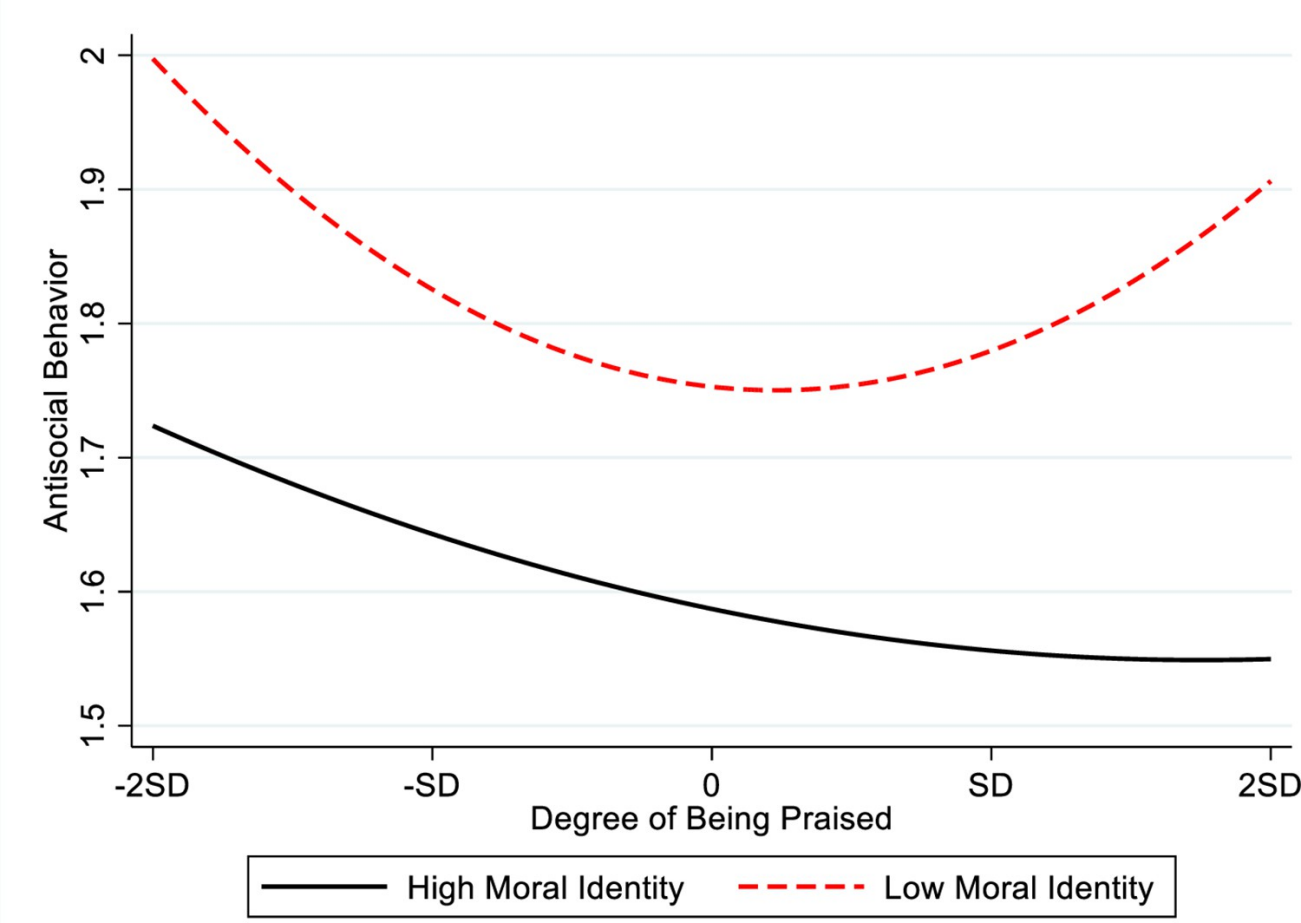
The moderating effect of moral identity on the curve relationship between praise and antisocial behavior.

The results of model 9 in Table 3 show that the interactive term between the square of the praise and moral identity is negatively related to antisocial behavior (β = -. 030, p < 0.05), which shows that moral identity moderates the relationship between the praise and antisocial behavior. In order to clearly display the moderating effect of moral identity on the curvilinear relationship between praise and antisocial behavior, the present study draws the moderating effect of moral identity according to the regression coefficient (Fig 5). Fig 5 shows that the impact pattern between praise and antisocial behavior is first falling and then rising with the increasing of the praise when the student’s moral identity is low; however, there is a stable negative relationship between the praise and antisocial behavior when the student’s moral identity is high. Similar to test Hypothesis 3a, we conduct the simple slope test to test whether the impact of praise on antisocial behavior is significant in different cases of moral identity, and the results are shown in Table 8. The results show that when the level of moral identity is high, the praise will reduce antisocial behavior when the praise is located at -SD and 0 (β = -.037, p < .050;β = -.029, p < 0.01). When the praise was located at the -2SD, 0, and 2SD, the effect of the praise on antisocial behavior was negative but not significant (β = -.046, p > .050; β = -.021, p > 0.05; β = -.012, p > 0.05). Therefore, in the case of a high level of moral identity, the praise will reduce antisocial behavior with the increase of the praise. In other words, no matter the level of praise, it will not promote the emergence of student antisocial behavior in the case of students with high moral identity. In the case of low level of moral identity, praise will significantly reduce antisocial behavior when the praise located at -2SD,-1SD, and 0 (β = -.192, p < .001; β = -.113, p < .001; β = -.035, p < .001); however, when the praise raise to the value of 1SD and 2SD, it was a significant positive related to antisocial behavior (β = .043, p < 0.01; β = .122, p < .001). In other words, in the case of a low level of student moral identity, the praise will be positively related to the antisocial behavior after it exceeds a certain degree, and the positive effect is stronger than the main effect of the praise (compared with the slopes at 1SD and 2SD in Table 5), thus, Hypothesis 3b is supported.

**Table 8:** Simple slope test of and praise*moral identity on antisocial behavior.

Adjustment variable	Academic achievement	Slope	Slope variance	T value
High moral identity	-2SD	-0.046	0.028	-1.618
	-SD	-0.037*	0.016	-2.268
	0	-0.029***	0.008	-3.722
	SD	-0.021	0.013	-1.602
	2SD	-0.012	0.024	-0.508
Low moral identity	-2SD	-0.192***	0.026	-7.252
	-SD	-0.113***	0.015	-7.805
	0	-0.035***	0.008	-4.648
	SD	0.043**	0.015	2.821
	2SD	0.122***	0.027	4.446

Note: * p < .015 ** p < .01, *** p < .001

### Robustness test

We use different data sources to conduct robust tests of our findings. Table 9 shows the regression results of using the data of student self-rated academic performance and self-rated antisocial behavior, Table 10 shows the regression results of using the data of parent-rated academic performance and parent self-rated antisocial behavior, Table 11 shows the regression results of using the data of parent-rated academic performance and parent-rated antisocial behavior. The results in Tables 9–11 show that the findings on the relationship between academic performance and antisocial behavior are robust.

**Table 9:** Regression analysis.

	Antisocial Behavior (student rated)								
	Model 1	Model 2	Model 3	Model 4	Model 5	Model 6	Model 7	Model 8	Model 9
Gender	-0.21***	-0.19***	-0.18***	-0.17***	-0.17***	-0.21***	-0.21***	-0.19***	-0.19***
Registration	0.01	0.02	0.02	0.02	0.02	0.01	0.01	0.01	0.01
One child/family	0.08***	0.06***	0.06***	0.05***	0.05***	0.08***	0.08***	0.06***	0.06***
Economic situation	-0.01	0.02*	0.03*	0.03**	0.03*	0.00	0.00	0.01	0.01
Parental relationship	0.07***	0.07***	0.06***	0.06***	0.06***	0.07***	0.07***	0.07***	0.07***
Relationship with father	-0.08***	-0.06***	-0.06***	-0.05***	-0.05***	-0.07***	-0.07***	-0.05***	-0.05***
Relationship with mother	-0.10***	-0.08***	-0.08***	-0.06***	-0.06***	-0.09***	-0.09***	-0.07***	-0.07***
AP		-0.15***	-0.16***	-0.12***	-0.12***				
AP^2^			0.09***	0.09***	0.09***				
DBP						-0.09***	-0.09***	-0.07***	-0.06***
DP22							0.06***	0.06***	0.06***
Moral identity				-0.18***	-0.16***			-0.20***	-0.17***
AP*MI					-0.01				
AP^2^*MI					-0.04**				
DBP*MI									0.01
DBP^2^*MI									-0.03*
R^2^	0.09	0.10	0.12	0.14	0.15	0.10	0.10	0.13	0.14
F	114.66***	126.79***	122.00***	143.03***	120.00***	108.84***	101.01***	128.13***	107.29***

Note: * p < .05, ** p < .01, *** p < .001; Academic performance is rated by students, AP = Academic performance, DBP = Praise

**Table 10:** Regression analysis.

	Antisocial behavior (parent rated)								
	Model 1	Model 2	Model 3	Model 4	Model 5	Model 6	Model 7	Model 8	Model 9
Gender	-0.19***	-0.16***	-0.16***	-0.15***	-0.15***	-0.19***	-0.19***	-0.18***	-0.18***
Registration	0.01	0.01	0.01	0.01	0.01	-0.01	0.01	0.01	0.01
One child/family	0.12***	0.11***	0.12***	0.11***	0.11***	0.12***	0.11***	0.11***	0.11***
Economic situation	-0.02	-0.02	-0.01	-0.01	-0.01	-0.02	-0.01	-0.01	-0.01
Parental relationship	0.05***	0.05***	0.05***	0.05***	0.05***	0.05***	0.05***	0.05***	0.05***
Relationship with father	-0.06***	-0.05***	-0.065***	-0.04***	-0.04***	-0.06***	-0.06***	-0.05***	-0.04***
Relationship with mother	-0.10***	-0.09***	-0.09***	-0.07***	-0.07***	-0.09***	-0.09***	-0.08***	-0.08***
AP		-0.18***	-0.16***	-0.16***	-0.15***				
AP^2^			0.05***	0.04***	0.03**				
DBP						-0.06***	-0.06***	-0.04***	-0.04***
PDBP^2^							0.04***	0.04***	0.04***
Moral identity				-0.11***	-0.08***			-0.12***	-0.10***
AP*MI					0.04***				
AP^2^*MI					-0.04*				
DBP*MI									-0.01
DBP^2^*MI									-0.03*
R^2^	0.08	0.11	0.11	0.12	0.13	0.08	0.08	0.09	.10
F	100.47***	125.86***	113.47***	113.67***	97.25***	91.56***	82.63***	88.24***	74.01***

Note: * p < .05, ** p < .01, *** p < .001; Academic achievements are rated by parents, AP = Academic performance, DBP = Praise

**Table 11:** Regression analysis.

	Antisocial behavior (self-rated)								
	Model 1	Model 2	Model 3	Model 4	Model 5	Model 6	Model 7	Model 8	Model 9
Gender	-0.21***	-0.19***	-0.19***	-0.17***	-0.17***	-0.21***	-0.21***	-0.19***	-0.19***
Registration	0.01	0.01	0.01	0.01	0.02	0.01	0.01	0.01	0.01
One child/family	0.09***	0.09***	0.19***	0.08***	0.08***	0.09***	0.08***	0.07***	0.07***
Economic situation	-0.01	-0.01	-0.01	0.01	0.01	-0.01	-0.01	0.01	0.01
Parental relationship	0.07***	0.07***	0.07***	0.07***	0.07***	0.07***	0.07***	0.07***	0.07***
Relationship with father	-0.08***	-0.07***	-0.07***	-0.05***	-0.04***	-0.07***	-0.07***	-0.05***	-0.05***
Relationship with mother	-0.10***	-0.10***	-0.10***	-0.07***	-0.07***	-0.09***	-0.09***	-0.07***	-0.07***
AP		-0.15***	-0.14***	-0.12***	-0.12***				
AP^2^			0.03*	0.02*	0.02*				
DBP						-0.10***	-0.10***	-0.08***	-0.07***
DBP^2^							0.05***	0.05***	0.06***
Moral identity				-0.19***	-0.17***			-0.20***	-0.17***
AP*MI					0.01				
AP^2^*MI					-0.03*				
DBP*MI									-0.02
DBP^2^*MI									-0.04*
R^2^	0.08	0.11	0.12	0.14	0.15	0.09	0.10	0.13	.14
F	120.19***	132.03***	117.95***	142.54***	97.25***	116.39***	106.40***	133.12***	111.93***

Note: * p < .05, ** p < .01, *** p < .001; Academic performance are rated by parents, AP = Academic performance, DBP = Praise

## Discussion

### Theoretical implications

This study at least has three theoretical implications. First, this study can help us to understand the relationship between academic performance and antisocial behavior by testing the U-shaped effect of academic performance on antisocial behavior. Most of the existing research finds that academic performance can reduce antisocial behavior by improving students’ self-esteem [21]. The current study discovered that academic performance cannot consistently decrease antisocial behavior; in fact, it may escalate antisocial tendencies when academic performance surpasses a certain threshold. The findings of the present study are partially consistent with existing research, indicating that academic performance, within a certain range, can have a negative impact on antisocial behavior [2, 30]. Furthermore, this study also reveals that academic performance, when exceeding a certain threshold, can positively predict antisocial behavior. This expands upon the conclusions of previous research findings. This discovery aids in effectively explaining the campus phenomenon where an "excellent student" might also exhibit "antisocial behavior". This study proposes that students with excellent academic performance may decrease their moral standards and form psychological pride and entitlement, and such perceptions and psychological status are effective predictors of antisocial behavior [44]. This finding explains why many students with excellent performance can be observed in deviant behavior. The results of this study are conducive to expanding our understanding of the effect of academic performance on antisocial behavior through testing the curve relationship between academic performance and antisocial behavior.

Second, the findings of the present study can deepen our understanding on how student’s academic performance influences their antisocial behavior by examining the curvilinear mediating mechanism of praise. On one hand, our research reveals a U-shaped relationship between praise and antisocial behavior, aligning partially with previous studies. Specifically, within a certain range, we observed that praise can decrease students’ antisocial behavior. This finding is in line with previous research indicating that being praised enhances students’ self-efficacy and self-esteem to a certain extent [40] and reduces antisocial behavior [21]. Further, this study also found that excessive praise may bring negative effects on antisocial behavior, that is, when the degree of praise exceeds a certain level, it will promote students to conduct antisocial behavior. On the other hand, this study also found the curvilinear relationship between academic performance and antisocial behavior is mediated by praise. It means that the better the academic performance, the higher the degree of praise, which will in turn influence antisocial behavior. According to the moral licensing theory, when individuals’ achievements meet social expectations, they will decrease their moral requirements, and then they may conduct antisocial behaviors [19]. Hence, the findings of this study offer empirical support for the moral licensing theory. According to this theory, when students attain outstanding academic outcomes, they receive more praise from teachers, leading them to develop a sense of entitlement, potentially lowering their moral standards and consequently engaging in antisocial behavior. The examination of the indirect effect of academic performance on antisocial behavior through praise can deepen the understanding of how academic performance impacts antisocial behavior.

Third, this study found that excellent performance and excessive praise do not necessarily engage in antisocial behavior, but whether the student conducts antisocial behavior also depends on their moral identity. Specifically, when the level of student moral identity is high, academic performance and praise are negatively correlated with antisocial behavior; However, when the level of individual moral identity is low, if academic performance and praise exceed a certain threshold, it will promote student conduct antisocial behavior. The conclusion drawn in this study aligns with previous findings related to moral identity. Prior research indicates that individuals with a high moral identity tend to hold themselves to stringent internal moral standards when making behavioral decisions. They actively inhibit thoughts and actions that conflict with these moral standards, thereby ensuring consistency between their behavior and their moral values. [37, 38]. When individuals have a low level of moral identity, they are less likely to base their behavioral decisions on their moral schema [37]. In such scenarios, individuals possessing a sense of moral permission or privilege tend to feel more at ease when indulging in antisocial behaviors. The findings of this study contribute significantly to a deeper understanding of the impact of academic achievement and praise on antisocial behavior.

### Practical implications

First, the administrator needs to pay attention to the "moral loose" brought by academic performance to avoid junior students’ antisocial behavior. The empirical finding of this study can help school administrators and teachers identify the moral issues brought on by academic performance [48], that is, while affirming students’ academic performance, we usually tend to acquiesce them are in a high moral level and ignore the possibility of the fact that students with excellent performance are likely to violate social norms. The administrators and teachers who attach great importance to academic achievements should pay attention to this potential "moral loose". Because students with excellent academic performance may misdiagnose and think that doing something that wanders on the edge of morality will not bring punishment to themselves [49]. Therefore, the findings of this study can provide educational practitioners with management suggestions to help them realize both the positive and negative effects of academic performance. For example, while they encourage students to improve their academic performance, educational administrators should establish appropriate mechanisms to monitor antisocial behaviors to ensure that excellent students’ behaviors do not exceed moral boundaries [50].

Second, the administrators should appropriately take advantage of praising to avoid the negative effect of excessive praise. Based on the CEPS survey of junior school students, this study found that a certain level of praise can effectively reduce antisocial behavior; however, if the degree of praise exceeds a threshold, it will encourage students to conduct antisocial behavior [30]. Therefore, while the administrator and teachers pay attention to the positive role of praise in the teaching process, they should not ignore the negative impact of excessive praise on students. Specifically, teachers should be good at applying praise in the classroom, for example, they can adopt the approach of "encouraging and guiding" to give students appropriate praise [28]. However, they should avoid being praised for students with excellent academic performance too frequently, which will cause them to breed some negative psychology, and then lead to antisocial behavior.

Third, the administrator and teacher need to pay effort to improve junior high school students’ moral standards, for example, improving the level of moral identity. This study found that moral identity is a boundary condition on the relationship between academic performance, praise, and antisocial behavior [2]. If students have a high level of moral identity, no matter the level of academic performance and praise, it will not promote student engagement in antisocial behavior; however, if students’ moral identity level is low, excellent academic performance and excessive praise will lead to students’ antisocial behavior [21]. Therefore, for school administrators and teachers, they should strengthen students’ moral education and improve students’ level of moral standards. For example, increasing the level of students’ moral standards can not only rely on the moral courses but also embed the moral disciplines in school culture [6, 51].

### Research limitations and future directions

The present study inevitably has several limitations. First, students’ academic performance in this study is collected through subjective evaluation. Although subjective measurement error may influence the findings of this study, the robust test of this study shows that this error has little potential influence on our conclusions. Future research can use students’ actual and objective scores to test the relationship between academic performance and antisocial behavior. Secondly, due to the limitation of the data, academic performance, praise, moral identity, and antisocial behavior, all come from the second wave data; therefore, it is impossible to infer the causal relationship between the three variables. Future research can use a longitudinal design to collect data to test the relationship between academic performance and antisocial behavior. Finally, individual antisocial behavior is also closely related to individual personality, however, we didn’t control these factors that may potentially impact the generalization of our findings. Future research can further control individual personality (e.g., egoism) to test the relationship between academic performance and antisocial behavior.

## Conclusions

This study found that junior school students’ academic performance is U-shaped related to antisocial performance, specifically, junior school student with a high level of academic performance is positively related to their antisocial behavior, and the praise mediates the curvilinear relationship between academic performance and antisocial behavior. Furtherly, this study found that moral identity moderates the U-shaped relationship between academic performance and antisocial behavior, specifically, there is a U-shaped relationship between academic performance and antisocial behavior when the student’s moral identity is low, and there is a negative relationship between academic performance and antisocial behavior when the student’s moral identity is high.

## Supporting information

S1 Data (ZIP)
